# Personal

**Published:** 1908-07

**Authors:** 


### PERSONAL.

Dr. William C. Gorgas, assistant surgeon general of the United States army, supervisor of sanitation in the Panama canal zone, whose official address is Ancon, Panama, was chosen to be president-elect of the American Medical Association at its meeting held at Chicago, June 2-5, 1908. The conspicuous services rendered by Dr. Gorgas as chief sanitary officer of Havana and later in a similar manner on the canal zone places him in the forefront of sanitarians. His election to this high office is in effect a tribute to the great work he has accomplished.

Dr. Charles G. Stockton, of Buffalo, was appointed a member of the committee of arrangements for the United States of the International Medical Congress of 1909, to be held at Budapest.

Dr. Alvin A. Hubbell, of Buffalo, was elected chairman of the section on ophthalmology of the American Medical Association, during the recent meeting held at Chicago.

Dr. Grover W. Wende, of Buffalo, was appointed a member of the committee of arrangements for the United States of the International Medical Congress of 1909, to be held at Budapest.

Dr. Wisner R. Townsend, of New York, secretary of the Medical Society of the State of New York, was elected a member of the board of trustees of the American Medical Association at the Chicago meeting.

Dr. A. L. Benedict, of Buffalo, now making a tour of Europe, has gone from England to Paris, where he will spend some time in visiting places of interest and then further extend his tour to the interior of the continent.

Dr. Walter B. Chase, of Brooklyn, has been elected president of the council of the Long Island College Hospital.

Dr. Walter P. Manton, of Detroit, was elected chairman of the section on obstetrics and diseases of women of the American Medical Association at the recent meeting in Chicago.

Dr. John H. Grant, of Buffalo, has removed his residence from the Hotel Rockford to The Cutler, 380 Franklin Street.

Dr. John H. Pryor, of Buffalo, has been appointed by Dr. Eugene H. Porter, state commissioner of health, one of the lecturers on tuberculosis recently authorised by the state civil service commision.

Dr. Thomas Bagley, of Buffalo, has been appointed a member of the board of trustees of the Blocher Home at Williamsville, N. Y.

Dr. DeWitt C. Greene, of Buffalo, has been appointed a member of the board of trustees of the Blocher Home at Williamsville, N. Y.

